# PRC1 and PRC2 Are Not Required for Targeting of H2A.Z to Developmental Genes in Embryonic Stem Cells

**DOI:** 10.1371/journal.pone.0034848

**Published:** 2012-04-09

**Authors:** Robert S. Illingworth, Catherine H. Botting, Graeme R. Grimes, Wendy A. Bickmore, Ragnhild Eskeland

**Affiliations:** 1 MRC Human Genetics Unit, MRC Institute of Genetics and Molecular Medicine at the University of Edinburgh, Edinburgh, United Kingdom; 2 BMS Mass Spectrometry and Proteomics Facility, Biomedical Sciences Research Complex, University of St. Andrews, St. Andrews, Fife, United Kingdom; CNRS, France

## Abstract

The essential histone variant H2A.Z localises to both active and silent chromatin sites. In embryonic stem cells (ESCs), H2A.Z is also reported to co-localise with polycomb repressive complex 2 (PRC2) at developmentally silenced genes. The mechanism of H2A.Z targeting is not clear, but a role for the PRC2 component Suz12 has been suggested. Given this association, we wished to determine if polycomb functionally directs H2A.Z incorporation in ESCs. We demonstrate that the PRC1 component Ring1B interacts with multiple complexes in ESCs. Moreover, we show that although the genomic distribution of H2A.Z co-localises with PRC2, Ring1B and with the presence of CpG islands, H2A.Z still blankets polycomb target loci in the absence of *Suz12, Eed* (PRC2) or *Ring1B* (PRC1). Therefore we conclude that H2A.Z accumulates at developmentally silenced genes in ESCs in a polycomb independent manner.

## Introduction

Genome-wide mapping relative to cis-regulatory elements and transcriptionally active and repressed loci has implicated histone modifications in gene regulation. Another mechanism of chromatin regulation is the replacement of core histones with histone variants. Unlike the S-phase restricted expression of canonical histones, histone variants are expressed throughout the cell cycle. They have been implicated in the regulation of transcription, DNA repair, chromosome segregation and spermatogenesis [1]. Variant histone H2A.Z is essential and has been linked to both transcriptional activation and repression [2]. In *Drosophila* embryos [3] and human CD4+ T cells [4], H2A.Z is enriched around the transcription start site (TSS) of active genes and it is incorporated into promoters of estrogen-receptor target genes upon their induction by estrogen [5]. Consistent with a link to active chromatin, TSS-proximal H2A.Z containing nucleosomes are enriched in H3K4me3 and depleted in H3K9 methylation [4]. In *Saccharomyces cerevisiae* H2A.Z is at promoters of both active and inactive genes [6] and prevents the spreading of telomeric heterochromatin into euchromatin [7], [8].

Despite the association with transcriptional activity, *Drosophila* H2A.Z (H2Av) is also involved in the establishment of centromeric heterochromatin. It co-localises with Heterochromatin protein 1 alpha (HP1α) at pericentric heterochromatin in some mouse cells [9], [10] and it has been suggested to co-operate with HP1α to stabilise compact chromatin [11], [12].

H2A.Z/H2Av is also implicated in polycomb-mediated gene silencing in *Drosophila* [11] and in mouse embryonic stem cells (ESCs). H2A.Z occupies a similar set of silent developmental genes as the polycomb repressive complex 2 (PRC2) component Suz12 [13]. PRC2 trimethylates histone H3 lysine 27 (H3K27me3) through the action of the histone methyltransferases (HMTases) EZH1/2 [14]. This creates a binding platform for recruitment of the PRC1, which contains the E3 ligases Ring1A/B that can monoubiquitinate histone H2AK119 (H2AK119ub1) [15]. Moreover, Ring1B can also monoubiquitinate H2A.Z at several lysine residues in the C-terminal tail, including K120 [9]. H2A.Zub is enriched on the inactive X chromosome of female mammalian cells indicating a link with facultative heterochromatin [9].

PRC2 has been suggested to be necessary for H2A.Z incorporation into silent genes in ESCs and, reciprocally, H2A.Z knockdown was reported to impair PRC2 binding and gene repression at polycomb-target loci [13]. This suggested some mutual dependency between H2A.Z and polycomb.

Exchange of H2A/H2B dimers with H2A.Z/H2B is mediated by the SWR1 ATP-dependent chromatin remodelling complexes (Tip60-p400.com, SWR1-C and NuA4 complex) [16], [17], [18], [19]. Tip60-p400.com is important in maintaining ESC identity and repression of developmental genes, and the p400 ATPase subunit is detected at most PcG target genes [20].

How H2A.Z deposition is targeted to polycomb genes in ESCs, and whether it is dependent on PRC1 as well as PRC2, are not known. To address these questions, we identified Ring1B-associated proteins in ESCs and did find components of p400.com. We show that H2A.Z co-localizes with H3K27me3, EZH2 and Ring1B at developmentally silenced gene loci in ESCs. However, using ESCs mutant for Suz12, Eed (PRC2) or Ring1B (PRC1), we show that H2A.Z enrichment at these genes is not dependent on either PRC2 or Ring1B (PRC1). Rather we find that H2A.Z occupancy correlates well with the presence of CpG islands.

## Results

### Ring1B is Associated with known Polycomb Complexes in ESCs

In somatic cells, proteomic analysis has identified that Ring1B is present in several complexes including PRC1, BcoR and E2F6.com [21], [22], [23], [24], [25], however a full analysis of Ring1B partners has not been examined in ESCs.

**Figure 1:**
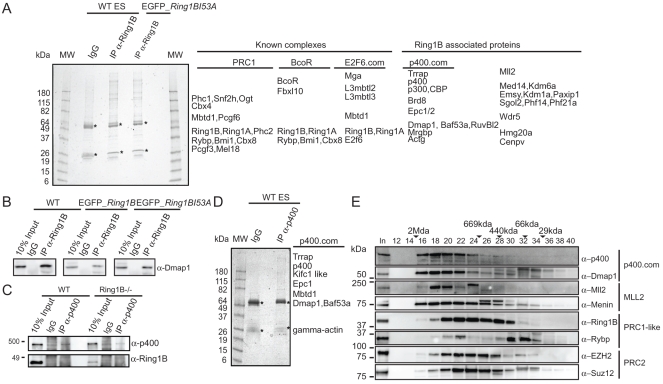
Identification of Ring1B associated peptides in mouse ESCs. A) IPs of Ring1B from nuclear extracts of WT and *Ring1B^−/−^* cells rescued with Ring1BI53A, resolved by 4–20% SDS-PAGE and stained with colloidal blue. Proteins that had a significant number of detected peptides by mass spectrometry analysis are listed. Peptides also detected in the control IgG lane were subtracted. Asterisks indicate the heavy and light Ig chains. B) IP of Ring1B from nuclear extracts of WT and *Ring1B^−/−^* cells rescued with full length Ring1B or Ring1BI53A resolved by 12% SDS-PAGE and immunoblotted with α-Dmap1. C) Immunoblot of p400 IP from WT and *Ring1B^−/−^* ESCs using p400 and Ring1B antibodies. D) IP of endogenous p400 from nuclear extracts of WT ESCs resolved by 4–20% SDS PAGE. Polypeptides stained by colloidal blue were identified by mass spectrometry. Listed are proteins with significant number of peptide hits. E) Size exclusion chromatography of nuclear extract from WT ESCs. Every other fraction (50%) were resolved by 4–15% SDS-PAGE and immunoblotted with anti-p400, anti-Dmap1, anti-Mll2, anti-Menin, anti-Ring1B, anti-Rybp, anti-Ezh2 and anti-Suz12. The upper panel represent fraction number, Input (In), black triangles indicate the retention of molecular weight standards in mega Dalton (MDa) or kilo Dalton (kDa).

We have previously shown that PRC1 components Phc2, Mel18 and Rybp are immunoprecipitated (IP’d) with Ring1B in mouse ESCs, while PRC2 component Ezh2 did not interact [26]. To determine the more complete Ring1B interaction network, we IP’d endogenous Ring1B from ESC nuclear extract and identified the associated proteins by mass spectrometry (nLC-ESI-MS/MS) (Fig. 1A and Table S1). Amongst the associated proteins we found: Phc1/2, Snf2h, Ring1A, Rybp, Pcgf3/6, Bmi1, Mel18 and Cbx4/8 - all previously found to be components of, or associated with, PRC1 [27]. Moreover, we identified Ogt whose *Drosophila* homologue *O-*GlcNAcylate Polyhomeotic and is involved in polycomb repression [28], [29].

In somatic cells, Ring1B was also previously co-purified as a component of the Bcl6 co-repressor complex (BcoR) [21], [22] which has a role in regulating gene expression during early ectoderm and mesoderm differentiation [30]. We found BcoR itself and the lysine demethylase Fbxl10 co-purifying with Ring1B. We also identified H3K4 demethylase Kdm1a/Lsd1 that interacts with BcoR in somatic cells [22], but we did not identify NsPC1/Pcgf1.

We further identified major components of the E2F6.com repressive complex: E2F.6, Mga, Mbtd1, L3mbtl2/3 [31]. L3MBTL2 has recently been described in a PRC1-like complex containing RING1A, RING1B, PCGF6 (MBLR), E2F6 and HP1g, and is involved in repression of a different set of targets from PRC2 [32].

Unexpectedly we also identified polypeptides for components of the Mll2 complex; Mll2, Wdr5, Paxip1 and Kdm6a, as well as HMG20a/b which are known to act as recruiters of the Mll complex [33]. There were a number of other proteins identified that were not previously known to interact with Ring1B (Fig. 1A, Table S1 and S2).

We have previously shown that I53A mutant Ring1B is unable to ubiquitinate H2A in vivo, but is nevertheless able to interact with other PRC1 components and is able to, compact the chromatin, and repress the expression, of Hox loci in ESCs [26]. nLC-ESI-MS/MS analysis of the I53A Ring1B IP revealed exactly the same protein complexes as for wild-type (WT) Ring1B (Fig. 1A, Table S2.) indicating that, as predicted *in vitro* [34], mutation of the I53 surface residue does not interfere with the incorporation of Ring1B into stable protein complexes.

### Ring1B Interacts with Subunits of p400.com

Interestingly, we identified most subunits (Trrap, p400, Epc1, Brd8, Dmap1, Ruvbl2, Baf53a, Mrgbp and actin) of p400.com [19], in the Ring1B IPs from ESC nuclear extracts (Fig. 1A and Tables S1 and S2). Benzonase nuclease treatment of the extract prior to IP confirmed that the Ring1B/p400 interaction is not dependent on nucleic acids (Table S3). Dmap1 had previously been found to interact with Bmi1 [35] and we show the Dmap1 interaction with Ring1B by immunoblot in WT and Ring1B-rescued cell lines (Fig. 1B). Dmap1 also co-fractionates with Ring1B in high molecular weight fractions in a sucrose-sizing gradient (Fig. S1). Ring1B fractionates with a broad profile supporting our finding that it is a constituent of multiple complexes in ESCs.

The human p400.com has previously been purified without the histone acetyltransferase (HAT) subunit Tip60/Kat5 associated [36] and we did not detect Tip60 in our Ring1B IPs. Formally, we cannot exclude that Tip60 is present as it has a similar size to the IgG heavy chain, which was omitted from the M/S analysis. We did however identify the HATs p300 and CBP, known to interact with p400.com in HeLa cells [36]. Nevertheless, we were not able to detect Ring1B by immunoblot or nLC-ESI-MS/MS in an IP for p400 (Figs. 1C, 1D and Table S4). Therefore, we suggest that p400 does not interact directly with Ring1B, but can associate with other components of Ring1B-containing complexes.

To further address the potential interaction of Ring1B with components of p400 and MLL2 complexes we performed size exclusion chromatography. Nuclear extract from WT ESCs was treated with RNase A and Benzonase nuclease, and separated on a Superose 6 column (Fig 1E). As a control we probed for PRC2 components Ezh2 and Suz12 and show that they co-fractionates in a broad peak (fractions 16–32). Ring1B shows a broad elution profile (fractions 16–30), consistent with sucrose gradient sedimentation data (Fig. S1), indicating participation in multiple PRC1- like complexes (Fig 1E). Rybp display a low molecular peak (fraction 28–34) and co-elutes with Ring1B in fractions 20–22. We observe that p400 and Dmap1 co-fractionates in high molecular weight complexes (440 kDa–2 MDa, fractions 16–28). The MLL component Menin is also found in fractions with molecular size from 440 kDa and 2 MDa, and peaks with Mll2 in fractions 18–20. Ring1B overlaps with p400, Dmap1 and Mll2 over several fractions, however, the main peaks of p400.com and Mll2 is at a larger molecular weight. We therefore suggest that Ring1B is not a stable component of these two complexes.

### H2A.Z Occupies Polycomb Targets in Ring1B Deficient ESCs

The presence of p400.com components in our Ring1B IPs prompted us to re-examine the relationship between H2A.Z occupancy and polycomb. A previous study had shown that H2A.Z is enriched at silenced developmental gene loci in ESCs that are also occupied by the PRC2 protein Suz12 [13]. Furthermore, H2A.Z was perturbed at these sites in Suz12 deficient ESCs, but the influence of PRC1 was not examined. Therefore, we sought to determine whether loss of Ring1B (PRC1) disrupts H2A.Z deposition at polycomb targets in ESCs.

**Figure 2:**
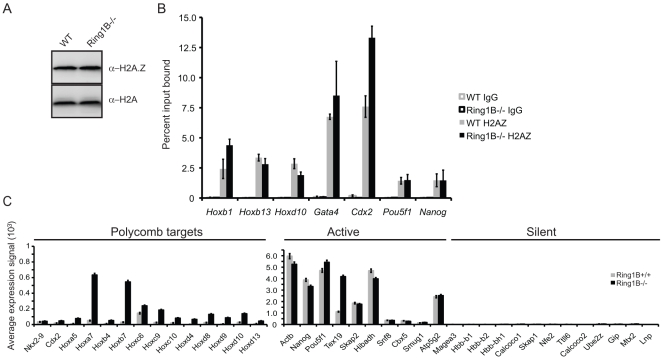
H2A.Z distribution at polycomb targets in the absence of Ring1B. A) Immunoblot for H2A.Z and H2A in *Ring1B^−/−^* and matched WT (Ring1B^+/+^) ESCs. B) ChIP for H2A.Z at the promoters of *Hoxb1, Hoxb13, Hoxd10, Cdx2, Pou5f1* and *Nanog,* assayed by qRT-PCR, in WT (*+/+*) (grey) or *Ring1B^−/−^* cells (black). Enrichment is shown as mean % input bound ± SD over two biological replicates (6 technical replicates). IgG is shown in white bars with grey or black borders ± SD. C) Average expression signals of active, silent and polycomb target genes in WT (+/+) (grey) and Ring1B*^−/−^* (black) from four biological replicates hybridized to MouseWG-6 v2.0 Expression BeadChips ± s.e.m.

We show that the H2A.Z antibody detect global levels of H2A.Z in WT ESC and calf thymus histones but did not recognize recombinant human H2A by immunoblot (Fig. S2A). Further we observed no change in the global levels of H2A.Z In Ring1B*^−/−^* ESCs compared to WT cells (Fig. 2A) [37]. Chromatin immunoprecipitation (ChIP) and real-time PCR (q-PCR) showed that, though occupancy by H2A.Z is marginally reduced at the promoter of *Hoxd10* in the absence of Ring1B, it is unaffected at *Hoxb13* and *Gata4*, and even increased, at the promoters of *Hoxb1* and *Cdx2* (Fig. 2B). The pluripotency genes *Nanog* and *Pou5f1* have only low levels of H2A.Z, which is above the IgG control background, and that is not affected in Ring1B mutant cells. Immunoblot of nuclear extract and q-PCR of Ring1B ChIP at the promoters of *Hoxb1*, *Hoxd1* and *Hoxd10* confirmed that Ring1B is absent in Ring1B*^−/−^* ESCs (Fig. S3A and B).

### EZH2 and H3K27me3 Coat Polycomb Target Loci in the Absence of Ring1B

We have previously shown that blanketing of H3K27me3 across the Hoxb and d loci is not grossly affected in the absence of Ring1B [26]. This is consistent, at least at these loci, with a model in which PRC1 acts downstream of PRC2, and PRC2 recruitment and maintenance does not solely depend on Ring1B-containing PRC1 activity [15], [38], [39].

**Figure 3:**
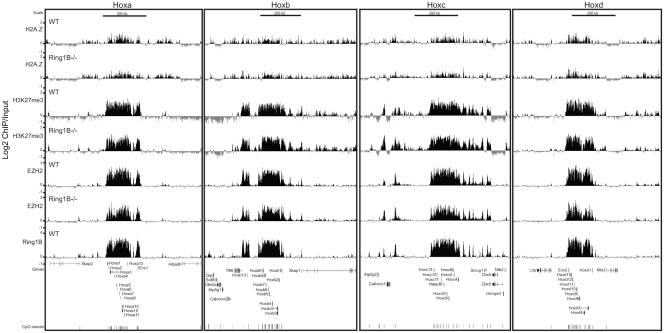
H2A.Z maps to Hox loci in wild-type and PRC1 mutant ESCs. Log2 of ChIP:input for: H2A.Z, H3K27me3, EZH2 and Ring1B from WT (+/+) (top) and *Ring1B^−/−^* (bottom) ESCs using a custom tiling microarray. Data is shown for the four paralogous murine hox loci (Hoxa, Hoxb, Hoxc and Hoxd) and their flanking genomic regions. The data represent a mean of 2 biological replicates. RefSeq gene annotations and CGIs are from the July 2007 (mm9) Build 37 assembly of the mouse genome (genome.ucsc.edu).

**Figure 4:**
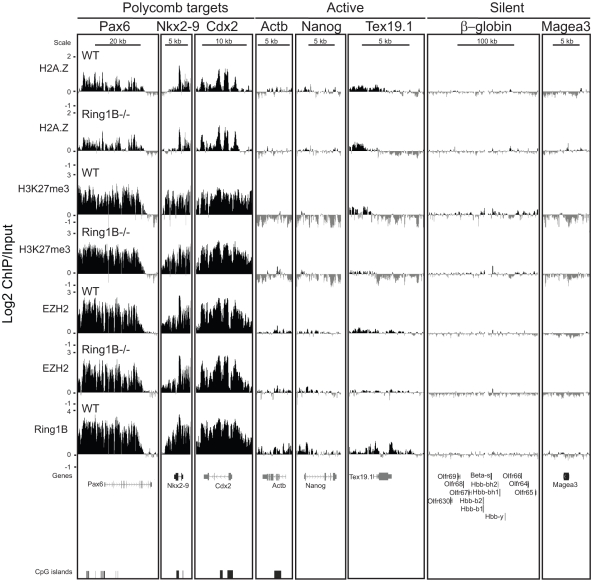
H2A.Z is enriched at developmental genes in wild-type and PRC1 mutant ESCs. Log2 of ChIP:input for: H2A.Z, H3K27me3, EZH2 and Ring1B from WT (+/+) (top) and *Ring1B^−/−^* (bottom) ESCs using a custom tiling microarray. Data is shown for selected polycomb target genes (*Pax6, Nkx2-9* and *Cdx2*), active genes (*Actb, Nanog* and *Tex19.1*) and silent genes (β-globin locus and *Magea3*). The data represent a mean of 2 biological replicates. RefSeq gene annotations and CGIs are from the July 2007 (mm9) Build 37 assembly of the mouse genome (genome.ucsc.edu).

To examine this more widely and to directly show binding of EZH2 in the absence of Ring1B, we performed ChIP on chip using a custom tiling array that covers many murine polycomb target loci, pluripotency genes and other non-polycomb target loci. Consistent with previous studies [40], [41], [42], [43], EZH2 and H3K27me3 specifically occupy all four Hox loci as well as the polycomb targets *Nkx2-9*, *Cdx2* and *Pax6* in WT ESCs. Ring1B occupies the same subset of developmental genes (Figs. 3 and 4) [42], [44], [45]. There is low polycomb enrichment over the highly transcribed *Nanog* and *β-Actin* [41] genes, but none at the silent *Magea3, β-globin* and surrounding olfactory receptor gene clusters that are not polycomb targets (Fig. 4) [46].

In the absence of Ring1B (*Ring1B^−/−^*) polycomb target genes are upregulated (Fig. 2C) [26], [44], [45], [47]. *Nkx2-9*, *Cdx2* and *Pax6* remain coated by H3K27me3 and EZH2 (Figs. 4) [26], [37], [45], consistent with Ring1B functioning downstream of PRC2. H3K27me3 and EZH2 also blanket the Hox loci in *Ring1B^−/−^* ESCs, albeit we observe some reduction in levels at specific regions: between *Hoxa1* and *2*, in the *Hoxa3* intronic region, between *Hoxb9* and *b13*, and at the *Hoxd4* promoter (Figs. 3).

### H2A.Z is Enriched at Promoters of Both Active and Silent Genes in the Absence of Ring1B

By ChIP on chip in WT ESCs we found that H2AZ occupancy at all four paralogous Hox loci and at the other polycomb targets examined overlaps with the distribution of H3K27me3, EZH2 and Ring1B. Moreover, H2A.Z remains at these regions in the absence of Ring1B (Figs. 3 and 4).

Consistent with our q-PCR data (Fig. 2B), but contrary to the study of Creyghton et al. [13], we also observe low, but significant, levels of H2A.Z by ChIP on chip at the promoters of the transcriptionally active *Nanog* and *Pou5f1* (Figs. 2B, C and 4). This discrepancy may be due to the better efficiency of the batch of H2A.Z antibody that we have used here (Fig. S2B, compare batches 170693 vs. 737918). H2A.Z also peaks around the TSS of highly transcribed genes (*Skap2, Hibadh, Atp5g1* and *Snf8)* that flank Hox loci (Figs. 3).

The germline specific gene *Tex19.1* is also expressed in ESCs [48] and its promoter and 5′end are enriched with H2A.Z (Fig. 4). There are low levels of EZH2 and H3K27me3 at the *Tex19.1* promoter and low levels of Ring1B both at the promoter, 5 and 3′ ends of the gene. Unlike most polycomb targets, levels of EZH2 and H3K27me3 are reduced at the *Tex19.1* promoter in the absence of Ring1B, suggesting that there is a dependency of PRC2 on Ring1B activity at this locus. Interestingly, *Tex19.1* expression is upregulated in *Ring1B^−/−^* ESCs (Fig. 2C) and this correlates with loss of H2A.Z around the TSS.

The silent β-globin genes and olfactory receptor gene clusters have no detectable H2A.Z, EZH2 or Ring1B at these loci (Fig. 4).

### H2A.Z Deposition in ESCs is not Dependent on PRC2 Function

**Figure 5:**
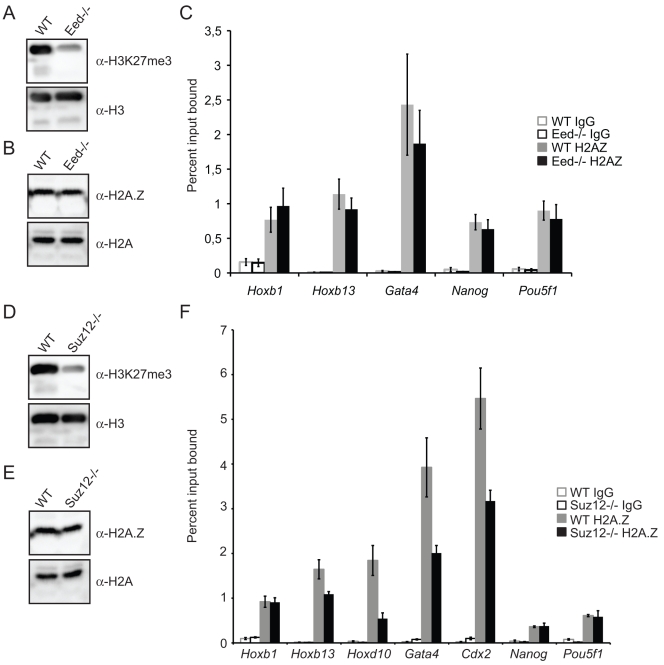
H2A.Z enrichment is not dependent on PRC2. A) Immunoblots for H3K27me3 and H3 in *Eed^−/−^* and matched WT ESCs B) Immunoblots for H2A.Z and H2A in *Eed^−/−^* and matched WT ESCs. C) ChIP for control IgG or H2A.Z at the promoters of *Hoxb1, Hoxb13, Gata4, Pou5f1* and *Nanog,* assayed by qRT-PCR, in WT (grey) or *Eed^−/−^* (black) ESCs. Enrichment is shown as mean % input bound ± s.e.m. over three biological replicates. IgG is shown in white bars. D) Immunoblots for H3K27me3 and H3 in *Suz12^−/−^* and WT (*Eed^+/+^)* ESCs E) Immunoblots for H2A.Z and H2A in *Suz12^−/−^* and WT (*Eed^+/+^)* ESCs. F) ChIP for control IgG or H2A.Z at the promoters of *Hoxb1, Hoxb13, Hoxd10, Gata4, Cdx2, Pou5f1* and *Nanog,* assayed by qRT-PCR, in WT (grey) or *Suz12^−/−^* (black) ESCs. Enrichment is shown as mean % input bound ± s.e.m. over three biological replicates. IgG is shown in white bars.

The general blanketing of H2A.Z at polycomb-regulated loci, even in Ring1B mutant cells, prompted us to re-examine the H2A.Z levels in cells lacking PRC2 function. Mutation of *Eed* leads to global loss of H3K27me3 in ESCs (Fig. 5A) [26], [41], [49], [50]. As observed for *Ring1B^−/−^* (Fig. 2A), immunoblotting of *Eed^−/−^* ESCs did not reveal a global loss of H2A.Z (Fig. 5B) [37]. Moreover, we observed no difference in H2A.Z levels by ChIP and q-PCR at the promoters of polycomb target loci (*Hoxb1*, *Hoxb13*, *Gata4*), or the promoters of the active *Pou5f1 and Nanog* between wild-type and *Eed^−/−^* cells (Fig. 5C). This was seen with different batches of H2A.Z antibody and by two independent observers R.S.I and R.E (Fig. 5C; 2n batch 170693 and 1n batch 737918; see also Fig. S2B).

Our finding that H2A.Z levels were unchanged in *Eed^−/−^* cells led us to investigate H2A.Z enrichment in *Suz12^−/−^* cells, used in the Creyghton et al [13]. Consistent with previous observations, global levels of H3K27me3 is lost in *Suz12^−/−^* ESCs (Fig. 5D) [51] and the global H2A.Z levels are unchanged (Fig. 5E). And as observed in *Eed^−/−^* cells, we found no change in H2A.Z levels by ChIP and q-PCR at promoters of *Hoxb1*, *Pou5f1 and Nanog* between wild-type ESCs (Fig. 5F). We did observe a slight decrease on the promoters of polycomb target loci (*Hoxb13*, *Hoxd10*, *Gata4* and *Cdx2*). However, we did not observe a drop to background levels as described for H2A.Z enrichment at the promoter of *Hoxb13* and other polycomb targets by Creyghton et al [13].

**Figure 6:**
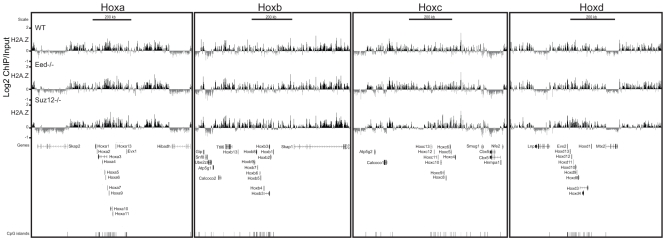
H2A.Z occupancy is not affected at Hox loci in PRC2 mutant ESCs. Log2 of ChIP: input for: H2A.Z from WT (+/+) (top) and *Eed^−/−^* (mid) and *Suz12^−/−^* (bottom) ESCs using a custom tiling microarray. Data is shown for the four paralogous murine hox loci (Hoxa, Hoxb, Hoxc and Hoxd) and their flanking genomic regions. The data represent a mean of 2 biological replicates, (3 technical replicates). RefSeq gene annotations and CGIs are from the July 2007 (mm9) Build 37 assembly of the mouse genome (genome.ucsc.edu).

**Figure 7:**
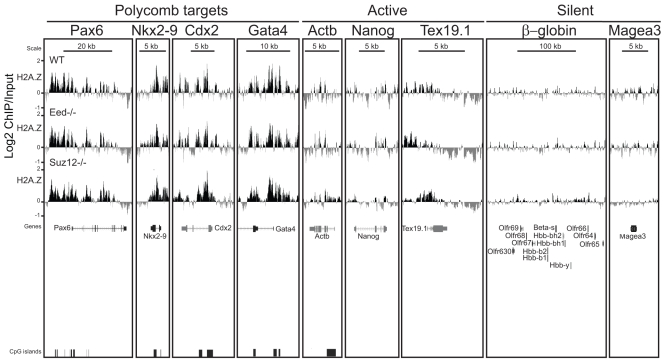
H2A.Z maps to developmental genes in wild-type *Suz12* *^−/−^* **and** ***Eed*** *^−/−^* **mutant ESCs.** Log2 of ChIP: input for: H2A.Z WT (+/+) (top), *Eed^−/−^* (mid) and *Suz12^−/−^* (bottom) ESCs using a custom tiling microarray. Data is shown for selected polycomb target genes (*Pax6, Nkx2-9, Cdx2* and *Gata4*), active genes (*Actb, Nanog* and *Tex19.1*) and silent genes (β-globin locus and *Magea3*). The data represent a mean of 2 biological replicates. RefSeq gene annotations and CGIs are from the July 2007 (mm9) Build 37 assembly of the mouse genome (genome.ucsc.edu).

To examine this more widely and to directly show binding of H2A.Z in the absence of PRC2, we did ChIP on chip on our custom tiling array (Figs. 6 and 7). H2A.Z ChIP on chip shows the same enrichment in both WT (*Ring1B^+/+^* and *Eed^+/+^*) ESCs, and we found that H2AZ occupancy at all four paralogous Hox loci and polycomb targets (Figs. 3, 4, 6 and 7). Moreover, H2A.Z blankets these regions in the absence of Eed or Suz12 (Figs. 6 and 7), and this was also true for the non-normalized datasets (data not shown). We did not observe a difference in H2A.Z enrichment over the active genes *Actb* and *Nanog.*

As we observed a decrease at promoters of *Hoxb13, Hoxd10, Gata4* and *Cdx2* by q-PCR in *Suz12^−/−^* cells (Fig. 5F), we performed pair wise scatter plot analysis of the different H2A.Z ChIP on chip datasets over +/− 1 kb of each arrayed TSS. The calculated correlation scores show that there is no difference between the two WT and three PRC mutant cells (Fig. S5).

**Figure 8:**
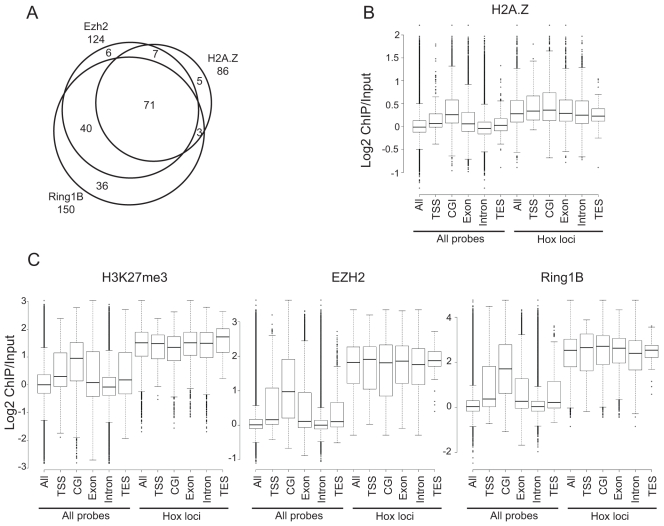
H2A.Z localises to CpG islands and overlap with EZH2 and Ring1B. A) Venn diagram showing overlap of genes displaying H2A.Z with EZH2 and Ring1B enrichment at TSS ± 500 bp within arrayed regions (total 240 unique TSS) in WT ESCs. B) Box plots showing Log2 H2A.Z ChIP/Input of over TSSs (±250 bp), CpG islands (CGI), exons, introns and transcription end sites (TES ± 250 bp) across all probes or Hox loci in WT ESCs. C) As in (E) but for EZH2, H3K27me3 and Ring1B.

The discrepancy of these data to the findings of Creyghton et al [13], led us to compare the distribution of H2A.Z, H3K27me3 and EZH2 across the genomic regions on our tiling array. There is an extensive overlap between H2A.Z, EZH2 and Ring1B across promoters (TSS +/− 0.5 kb) - H2A.Z bound TSSs overlaps 91% with EZH2 and 86% with Ring1B (Fig. 8A). However, we do not detect H2A.Z at approximately 30% of EZH2 and Ring1B bound TSS (Fig. 8A).

### H2A.Z Localises to CpG Islands

H2A.Z occupancy and DNA methylation are inversely correlated in plants and human B-cells [52], [53]. Polycomb target genes are generally associated with CpG islands (CGIs) that are, in the majority, unmethylated in ESCs [54]. CGIs have also been suggested to be important for recruiting polycomb complexes [42], [55], [56]. Indeed, we found that across the entire tiling array H2A.Z enrichment is higher at CGIs, than at TSS, exons, introns, or transcription end sites (TES) (Fig. 8B). Similarly, H3K27me3, EZH2 and Ring1B are most highly enriched at CGIs, followed by TSS and exons (Fig. 8C). Interestingly, this trend was not seen at the Hox loci. Here H3K27me3, EZH2, Ring1B and H2A.Z were highly enriched over all classes of sequence examined (Figs. 8B and 8C). We also noted that H2A.Z is absent from the CpG-rich promoter of *Magea3* that is regulated by Dnmt-mediated DNA methylation in ESCs (Fig. 4) [54].

We further compared the occupancy of EZH2 in WT versus Ring1B*^−/−^* cells, and found it was still present, though reduced, at CGIs, exonic and intronic sequences across the entire tiling array (Figure S4). H2A.Z enrichment also showed a smaller, but significant, decline at the same classes as EZH2 in *Ring1B^−/−^* cells (CGIs, exonic and intronic). There was not a significant decrease of EZH2 and H2A.Z over TSS and TES on all arrayed genes (Figure S4C and F). Together, our finding demonstrate that H2A.Z is highly enriched at CGIs in ESCs and that in the absence of Ring1B, H2A.Z occupancy is reduced to a lesser extent than EZH2 and still blankets polycomb target genes.

## Discussion

### Ring1B Interacts with Multiple Protein Complexes in ESCs

We have shown that Ring1B is part of several PRC1-like complexes in ESCs (PRC1 BCOR, PRC1L3, PRC1L4 and E2F6.com [21], [22], [23], [24], [25], [32]) and it will be important to dissect the respective functions of these different complexes at different target loci in ESCs.

We identified several subunits of the p400.com interacting with Ring1B, but we did not identify Ring1B or Tip60 in mass spectrometry analysis of the p400 IP. Biotin-tagging of Tip60 to map interactions in ESCs has previously identified the p400.com, but Ring1B was not co-purified [57]. We therefore suggest that p400 interact indirectly with Ring1B and that this may be through Dmap1 who has previously been found to interact with Bmi1 known to form heterodimer with Ring1B [35].

We identified Ogt in our Ring1B IP from ESCs nuclear extracts and this interaction was not affected by mutation of I53A mutant version of Ring1B that abolishes the ubiquitnation catalytic activity. *Drosophila* Ogt *O-*GlcNAcylates Polyhomeotic and is involved in polycomb repression [28]. Ogt has also been pulled down with Oct4 in ESCs, together with Ring1B, Phc1, Trrap, and p400 [58]. Furthermore, the Ph homolog Phc3 is *O-*GlcNAcylated in ESCs [59]. The role of *O-*GlcNAcylation mediated interactions needs to be explored further.

The transcription regulator Max is part of E2F6.com [31] and has recently been shown to interact with Ring1B, p400, Dmap1, Brd8, Tip60 and Trrap in ESCs [57]. This supports our finding that there may be a link between Ring1B, E2F6.com and p400.com. The *Drosophila* p400 homolog (Domino) interacts genetically with E2F and, together with MBT proteins, represses E2F target genes [60]. Moreover, Domino also represses *Hox* genes through a genetic interaction with PcG and TrxG [61].

We were surprised to find members of Mll2 complex in our Ring1B IP. However, this is not the first time TrxG proteins are identified together with PRC components. Ring1B has been previously been found in the MLL1-Wdr5 complex from Hela S3 cells [62] and in *Drosophila,* biotinylated Pc pulled down Ring1 and many Trx related proteins [63].

We used size exclusion chromatography of WT ESC nuclear extract to compare Ring1B elution profiles with IP’d components of p400 and MLL2 complexes (Fig 1E). Ring1B elutes with an apparent molecular weight of ∼200 kDa ∼1 MDa. Human PRC1 has an estimated size of ∼500 kDa [25], and our gel filtration chromatography confirms our IP data that Ring1B participate in multiple PRC1- like complexes. Although there is some overlap of Ring1B with p400 and MLL2 complex components, these complexes elute with a higher molecular weight (669 kDa–2 MDa), which indicates that Ring1B is not a stable component of these two complexes (Fig. 1E).

The identification of these novel interacting proteins of Ring1B may increase our understanding of its functions in multiple protein complexes in ESCs, and further studies are needed.

### Specific Sites of Ring1B-mediated PRC2 Recruitment

We saw that H3K27me3 and EZH2 mostly remained coating polycomb target genes in *Ring1B^−/−^* ESCs (Figs. 3 and 4). However, we did observe a general decrease of EZH2 across the Hox loci (Fig. S4A) and at some other specific sites. We conclude that the majority of EZH2 targeting is independent of Ring1B, but may be stabilized in the presence of PRC1-like complexes.

### H2A.Z Occupancy is not Dependent on Polycomb Complexes

Consistent with a previous report, we found that H2A.Z and polycomb (Ring1B, EZH2 and H3K27me3) co-occupy a common set of repressed developmental genes in ESCs (Figs. 3 and 4) [13]. This also compares well with another ChIP study showing p400 enrichment at the Hoxa locus, *Pax6* and *Nkx2-9* in ESCs [20]. We saw some enrichment of H2A.Z at the active *Actb* and *Nanog* genes, but not at the β-globin locus (Fig. 4) [20]. Moreover, H2A.Z is highly enriched at CGIs, as has also been observed for H3K27me3, EZH2 and Ring1B (Figs. 6B and C) [42], [55].

Contrary to the study of Creyghton et al [13], we conclude that the major targeting mechanism of H2A.Z to these genes is not dependent on either H3K27me3 (*Eed^−/−^),* a functional PRC2 complex (*Eed^−/−^*), or a functional PRC1 complex (*Ring1B^−/−^*). H2A.Z is still enriched at polycomb target genes in these Ring1B or PRC2 mutant ESCs and we only observed subtle changes in H2A.Z abundance by qRT-PCR and array analysis in mutant cells (Figs. 2B, 3, 4, 5B, and S4A-F). We also performed H2A.Z ChIP in the *Suz12^−/−^* cells that were used in the Creyghton et al. [13] and did not observe the same dramatic drop to background levels of H2A.Z enrichment at developmental gene promoter regions and found that H2A.Z still blankets polycomb targets across arrayed regions (Figs. 5F, 7, 8 and S5). Therefore we think it is more likely that the explanation of discrepancy lies in differences in the efficiencies of the H2A.Z antibodies used (Fig S2B).

RNAi knockdown of p400 and Tip60, but not H2A.Z, affects ESC pluripotency, suggesting that the functional role of the p400 complex in pluripotency is not through H2A.Z incorporation [20]. Moreover, Ring1B knockdown resulted in a flattened colony morphology that was not seen for H2A.Z RNAi [20]. If polycomb complexes were orchestrating H2A.Z incorporation we would expect to find H2A.Z on all polycomb bound TSS. However we find a sizeable proportion of Ring1B and EZH2 enriched TSS that is not enriched for H2A.Z (Fig. 8A). Unmethylated CGIs have been suggested to contribute to polycomb recruitment in ESCs [55]. Indeed, we find that H2A.Z is most enriched at CGIs, and it is also located at transcription start sites (TSS) and exons. We conclude that it will be important to dissect the role of CGIs in recruitment of H2A.Z in ESCs.

## Materials and Methods

### Tissue Culture

The ESC line OS25 was cultured as previously described [26]. Feeder dependent ESC lines; Clone36 (*Ring1B^+/+^*), *Ring1B^−/−^* [47], *Ring1B* rescued cell lines [26], *Suz12^−/−^* (SBE8) [51], *Eed* ^+/+^ clone 2.21 and *Eed* mutant (17Rn5-3354SB) (*Eed^−/−^* B1.3) [49] were grown on mitomycin C-treated primary embryonic fibroblasts (PEFs) derived from E12.5 mouse embryos, in DMEM (Invitrogen) supplemented with 15% foetal calf serum (FCS, Hyclone), 1,000 units/ml LIF, non-essential amino acids, sodium pyruvate, 2-mercaptoethanol, L-glutamine, and Penicillin/Streptomycin. ESCs were trypsinized and the PEFs allowed re-attaching to the tissue culture plastic for 2×30 minutes in LIF-containing medium, before preparation of chromatin, and nuclear extracts from ESCs.

### Nuclear Extract Preparation and Immunoprecipitation

Nuclear extract was prepared from 2−4×10^8^ ESCs according to [64] with the following modifications: After precipitation with 1/10th volume of 4 M (NH_4_)_2_SO_4_ and mixing for 20 min, the lysate was cleared by centrifugation at 45,000 rpm (235,000×*g*) in a TL-100 ultracentrifuge (Beckman, Mountain View, CA). The supernatant was dialysed against 3 changes of Buffer C (25 mM HEPES pH 7.6, 150 mM NaCl, 12.5 mM MgCl_2_, 0.1 mM EDTA, 10% v/v glycerol, 1 mM DTT and freshly added complete protease inhibitors) and flash frozen in liquid nitrogen. Protein concentrations were quantified by Bradford (Bio-Rad) and a total of 400 µg nuclear extract were incubated with 4 µg mouse or rabbit IgG (Santa Cruz, sc-2025 and sc-2027), anti-Ring1B (MBL, D139-3), or anti-p400 (A300-541A-1 Bethyl Laboratories, Inc.) and immunoprecipitated with 20 µl Protein G Sepharose (GE Healthcare) for 2 h at 4°C. After three washes with BC300 (25 mM HEPES pH 7.6, 300 mM NaCl, 1 mM MgCl_2_, 0.5 mM EGTA, 0.1 mM EDTA, 10% v/v glycerol, 1 mM DTT, 0.2 mM PMSF and complete protease inhibitors containing 0.05% v/v NP40) for 10 min each, and once with BC100 (0.05% v/v NP40), the bound proteins were boiled in SDS sample buffer and resolved on Novex Tris-Glycine 4–20% gels (Invitrogen). Gels were stained with Colloidal Blue Staining Kit (Invitrogen). Bands from Ring1B IPs and IgG control were cut for mass spectrometry analysis. Alternatively, the IPd materials were transferred to a Hybond^TM^-P membrane (GE Healthcare) for immunoblotting with anti-Ring1B and anti-p400 (Table S6).

### nLC-ESI MS/MS Analysis

In-gel tryptic digestion was performed as previously described [65]. The peptides were then separated using an UltiMate nanoLC (LC Packings, Amsterdam) equipped with a PepMap C18 trap & column, using a gradient of increasing acetonitrile concentration, containing 0.1 % formic acid (5–35% acetonitrile in 180 min respectively, 35–50% in a further 30 min, followed by 95% acetonitrile to clean the column). The eluent was sprayed into a Q-Star XL tandem mass spectrometer (ABSciex, Foster City, CA) and analysed in Information Dependent Acquisition (IDA) mode, performing 1 sec of MS followed by 3 sec MSMS analyses of the 2 most intense peaks seen by MS. These masses are then excluded from analysis for the next 60 sec. MS/MS data for doubly and triply charged precursor ions was converted to centroid data, without smoothing, using the Analyst QS1.1 mascot.dll data import filter with default settings. The MS/MS data file generated was analysed using the Mascot 2.1 search engine (Matrix Science, London, UK) against UniProt April 2009 (7966092 sequences) or NCBInr March 2010 (10530540 sequences) databases (Taxonomy, *Mus musculus)*. The data was searched with tolerances of 0.2 Da for the precursor and fragment ions, trypsin as the cleavage enzyme, one missed cleavage, carbamidomethyl modification of cysteines as a fixed modification and methionine oxidation selected as a variable modification. Moreover, the Mascot hits were considered valid with a protein score >50, except for known members of the PRC1 complex. Peptides identified in control IgG lane and specific IP lane were discarded. All proteins were checked against the UniProtKB database (uniprot.org). IgG heavy and light chains were omitted from the analysis, and therefore proteins migrating with a similar molecular size could not be identified.

### Size Exclusion Chromatography

For Ring1B complex analysis a Superose 6 (10/300 GL, GE healthcare) was calibrated with standards MW-GF-1000 (Sigma) in BC200 buffer. One mg of WT E14 ESC nuclear extract was incubated 5 minutes at room temperature with 10 µg RNaseA (Roche) and 10 µg Benzonase (Novagen) and passed through a 0.22 µm filter before it was loaded on the Superose 6 column. The column was run isocratically in BC200 buffer for 1.4 column volumes and 0.5 ml fractions were collected. Every other fraction were TCA precipitated, 50% of the fraction was separated on a 4-15% SDS-PAA gel (TGX, BioRad) and immunoblotted. The same blot was reprobed and stripped with Restore Western Blot stripping buffer using anti-Ring1B, anti-Rybp, anti-EZH2, anti-Suz12, anti-Menin, anti-MLL2, anti-Dmap1, and anti-p400 antibodies (Table S6). Two biological replicates were analysed.

### Sucrose Gradient

A sucrose gradient experiment was performed as previously described [66] with the following modifications: 10-50% (w/v) sucrose gradients were prepared in BC100 buffer (25 mM HEPES/KOH (pH 7.3), 100 mM NaCl, 1 mM MgCl_2_, 0.5 mM EGTA, 0.1 mM EDTA, 10% glycerol (v/v), 1 mM DTT, and 0.2 mM PMSF). The gradient was prepared using a Gradient Station model 153 (BioComp) set at 1 min: 50 sec/80.0 degrees/21 rpm. A 500 µL sample containing 1.5 mg WT ESC nuclear extract or 30 μg of individual standards (Sigma, MW-GF-1000) was loaded on top of the gradient. Gradients were left for 30 min at 4°C then spun in a SW41 rotor (Beckman) at 41 000 rpm for 28 h at 4°C. 0.5 mL fractions were collected and analyzed by 10% SDS-PAGE.

### Acid Extraction of Histones

Histones were extracted as described in [26]. Recombinant human H2A was expressed and purified from *Escherichia coli* BL21(DE3)pLysS as previously described [67], [68], and Calf Thymus histones (H 9250) were purchased from Sigma. Histones were separated on a SDS-15% or 18% polyacrylamide gel and transferred to a Hybond^TM^-P membrane (GE Healthcare). Membranes were probed with anti-H3, anti-H3K27me3, anti-H2A and anti-H2A.Z (Abcam, batch 170693 and 726779).

### Chromatin Immunoprecipitation

Cells were cross-linked in 1% formaldehyde at room temperature for 10 min and ChIP was performed as described in [45]. Antibodies used were; Ring1B (MBL, D139-3), EZH2 (Millipore 07-689) and H2A.Z (ab4174; Abcam, batches 170693, 726779 and 737918) (Table S6). The H2A.Z antibody was raised against C-SLIGKKGQQKT, corresponding to amino acids 116-126 of Human H2A.Z and does not detect monoubiquitinated H2A.Z [9]. ChIP was performed overnight (o/n) with 3 µg H2A.Z antibody and 90 µg chromatin. For polycomb ChIPs; 10 µg Ring1B or EZH2 antibody was incubated with 450 µg chromatin o/n. ChIP and input chromatin was decrosslinked at 65°C for 6 h, then treated with RNase A (Roche) at 37°C for 1 h, then with Proteinase K (Genaxxon) at 55°C for 2 h. DNAs were purified using the QIAquick PCR purification kit (Qiagen). Real-time PCR analysis was carried on the LightCycler 480 System using SYBR Green Master mix (Roche) and oligos that are described in Table S5. The real-time thermal cycler was programmed as follows: 5 min Hotstart; 44 PCR cycles (95°C for 10 sec, 55°C for 25 sec, 72°C for 10 sec). Native ChIP for H3K27me3 is described in [26].

### Microarray Amplification, Labelling, Design and Analysis

In total 10 ng of input and ChIP DNA was amplified using the WGA2 whole genome amplification kit according to manufacturer’s instructions (Sigma). Amplified material was labelled with Cy3 or Cy5 using random priming with dye-labelled random hexamers according to the NimbleGen ChIP-chip protocol (Roche). In total, 2 biological replicates with dye swaps were hybridized for 20 h and washed according to manufacturer’s protocol.

A custom 3x720K mouse tiling array (NimbleGen, Roche) containing 179,493 unique probes from the genomic regions, with each probe represented by 4 replicates was used. Mean and median probe spacing is 45 bases. Arrays were scanned on a NimbleGen MS 200 Microarray scanner (Roche) using a laser power of 100% and 2 µm resolution and TIFF images analysed using MS 200 Data Collection software to quantitate raw signal intensities. The mean signal of the replicates was used for data analysis. The data was analysed using the LIMMA package and Bioconductor/R (version 2.10.1) (r-project.org) following Protocol 43 (www.epigenesys.eu) for Lowess normalisation [69].

For distribution analysis of Ring1B, EZH2, RNAPII and H2AZ,_microarray probes were binned according to ENSEMBL gene annotation (build mm9) as either TSS associated (TSS +/− 250 bp), exonic, intronic or associated with the transcription end sites (TES; +/− 250 bp). Probes mapping to CpG islands (CGIs) was also included in the analysis. Significance values were calculated where applicable using a two-sided Wilcoxon Rank test.

To identify regions of significant ChIP enrichment we applied the upperBoundNull function from the bioconductor Ringo package to each set of normalised microarray data. The threshold value was used to identify significantly enriched probes for each ChIP on chip dataset. TSSs (1 kb centred on the TSS) with a minimum of 4 significant probes were considered bound and these were cross-compared between the ChIP-chip datasets as expressed in three-way Venn diagrams.

H2AZ distribution was compared between each cell line by generating pair wise scatter plots depicting the mean log2 ChIP/input ratios for all probes located +/−1 kb of each arrayed TSS. Pearson’s product-moment correlation scores for each comparison were calculated using the ‘cor’ function in R.

All datasets are accessible from the NCBI Gene Expression Omnibus (ncbi.nlm.nih.gov/geo/) through GEO Series accession number GPL13276.

### Expression Analysis

RNA was extracted from *Ring1B^+/+^* and *Ring1B^−/−^* ESCs using Trizol reagent (Invitrogen). The quality of the RNA was assayed using Bioanalyzer (Agilent). Four biological replicates were amplified using Illumina TotalPrep RNA Amplification Kit and 200 ng labelled RNA was hybridized to MouseWG-6 v2.0 Expression BeadChips according to manufactureŕs protocol (Illumina). Average bead signal normalised were normalised for four datasets.

## Supporting Information

Figure S1 **Size fractionation of Ring1B complexes.** Size fractionation of nuclear extract from wild type OS25 ESCs by sucrose gradient sedimentation analysis (10–50%). Fractions were analyzed by immunoblot for Ring1B and Dmap1. Arrows indicate fractions where protein markers with indicated molecular masses peaked.
(EPS)

Figure S2 **Characterization of the H2A.Z antibody.** A) Specificity of H2A and H2A.Z antibodies. Upper panel shows global histones from WT ESCs (2.5 µg), calf thymus histones (2.5 µg) and recombinant human histone H2A (500 ng and 1 µg) were separated by 18% SDS-PAGE and immunoblotted with anti-H2A and anti-H2A.Z (batch #726779) antibodies. Coomassie staining of the respective blots are shown in the lower panel. Asterisks indicate degraded H2A. B) qRT-PCR analysis at the promoters of *Hoxb13, Hoxb10* and *Pou5f1* in WT ESCs after ChIP for three different batches of anti-H2A.Z, H3K4me3 antibodies and IgG. Enrichment is shown as mean % input bound ± SD.
(EPS)

Figure S3 **Control of Ring1B mutant ESCs.** A) Forty micrograms of cytoplasmic- (CE) and nuclear- (NE) extract from WT and *Ring1B^−/−^* ESCs were separated by 4–15% SDS-PAGE and immunoblotted with anti-tubulin, anti-HP1α and anti-Ring1B. B) ChIP for control IgG or Ring1B at the promoters of *Hoxb1, Hoxd1* and *Hoxd10,* assayed by qRT-PCR, in WT (grey) or *Ring1B^−/−^* (black) ES cell lines. Enrichment is shown as mean % input bound ± SD over three technical replicates. Control IgG is shown in white bars with grey or black borders ± SD.
(EPS)

Figure S4 **H2AZ and EZH2 are similarly distributed within all arrayed regions in WT vs. Ring1B ESCs (Related to** **Figs. 3** **and** **4** **).** Box plots showing the (A) log2 ChIP/Input distribution of EZH2 and H2A.Z over all probes (All) in WT versus Ring1B*^−/−^* ESCs. B) as described in A) over transcriptional start sites (TSS). C) as described in A) over CpG islands (CGI). D) as described in A) over exons. E) as described in A) over introns. F) as described in A) over transcription end sites (TES).
(EPS)

Figure S5 **H2A.Z distribution comparisons by pair wise scatter plots.** Scatter plots representing mean log2 H2A.Z ChIP/input for all probes located +/−1 kb of each arrayed TSS in two WT ESCs versus *Ring1B^−/−^, Eed^−/−^* and *Suz12^−/−^* ESCs. Pearson correlations are shown in upper right panels.
(EPS)

Table S1 Polypeptides associated with Ring1B (Relates to Fig. 1).
(PDF)

Table S2 Polypeptides associated with Ring1BI53A (Relates to Fig. 1).
(PDF)

Table S3 Polypeptides associated with Ring1B in presence of 50 U Benzonase (Relates to Fig. 1).
(PDF)

Table S4 Polypeptides associated with p400 (Relates to Fig. 1).
(PDF)

Table S5 Primers for ChIP analysis (Relates to Figs. 2 and 5).
(PDF)

Table S6 Antibodies used for this study.
(DOC)
