# Supplementary material for: PRC1 and PRC2 Are Not Required for Targeting of H2A.Z to Developmental Genes in Embryonic Stem Cells
Source: PLoS One. 2012 Apr 9;7(4):e34848. doi: 10.1371/journal.pone.0034848 (PMC3322156; doi:10.1371/journal.pone.0034848)
Supplement: Table S1 — Polypeptides associated with Ring1B (Relates to Fig. 1). (PDF) [file pone.0034848.s006.pdf]

Supplemental Table 1. Polypeptides associated with Ring1B (Relates to Fig. 1).

NCBI Acc. Nr.	Protein	Gene name	Mass (Da)	Protein Score[1]	Coverage[2] (%)
gi|13775066	O-linked GlcNAc transferase[a]	Ogt	118159	93	7
gi|1490546	Polyhomeotic-like 1[a]	Phc1	106891	703	26
gi|39795654	Mbt domain containing 1[a]	Mbtd1	70923	182	15
gi|3220232	Polyhomeotic-like 2[a]	Phc2	47556	60	3
gi|28076973	Polycomb group ring finger 6[a]	Pcgf6	40365	214	37
gi|2239144	Ring finger protein 1A[a,b,c]	Ring1A	39892	302	11
**gi|26354897**	**RING finger protein 1B[a,b,c]**	**Ring1B**	**38107**	**1180**	**41**
gi|5381327	RING1 and YY1-binding protein[a,b]	Rybp	24743	273	34
gi|27085286	BCL-6 interacting corepressor isoform[b]	Bcor	194094	41	2
gi|37360622	F-box and leucine-rich repeat protein 10 isoform 3[b]	Fbxl10	151474	46	3
gi|3023934	Histone deacetylase 2[b]	Hdac2	55837	102	12
gi|148696030	MAX gene associated[c]	Mga	330371	284	13
gi|27734414	l(3)mbt-like 2[c]	L(3)mbt-like2	80045	204	2
gi|148687065	Transformation/transcription domain-associated protein[d]	Trrap	420980	238	8
gi|27348237	E1A-binding protein p400[d]	p400	334589	276	9
gi|74179958	Bromodomain containing 8[d]	Brd8	95515	89	11
gi|149249371	Enhancer of polycomb homolog 2[d]	Epc2	91767	100	21
gi|27532963	Enhancer of polycomb homolog 1[d]	Epc1	90925	109	12
gi|12805675	DNMT1 associated protein-1[d]	Dmap1	51543	138	19
gi|6755382	RuvB-like protein 2[d]	Ruvbl2	51252	102	26
gi|4001805	BRG1-associated factor 53A[d]	Baf53a	47913	487	33
gi|6425087	Gamma actin-like protein[d]	Actg1	44029	218	27
gi|30425250	Actin, beta-like 2[d]	Actbl2	42319	125	19
gi|74190672	Beta actin[d]	Actb	42080	292	40
gi|5803102	Mortality factor 4 like 1[d]	Morf4l1	37321	54	22
gi|84569914	MRG-binding protein[d]	Mrgbp	22478	142	18
gi|149266757	Histone-lysine N-methyltransferase Mll2[e]	Mll2	571899	235	4
gi|145587671	Nuclear receptor coactivator 6[e]	Ncoa6	220333	68	0

gi|33859492	Lysine-specific demethylase 6A[e]	Kdm6a	159240	253	19
gi|42734451	PAX-interacting protein 1[e]	Paxip1	120505	501	23
gi|74144611	Ash2-like[e]	Ash2l	66825	116	30
gi|26339944	Retinoblastoma binding protein 5[e]	Rbbp5	59745	392	27
gi|16554627	WD repeat domain 5[e]	Wdr5	37136	426	41
gi|21313594	PTIP-associated 1[e]	Pa1	27820	64	15
gi|115270972	Mediator complex subunit 14	Med14	159966	77	5
gi|20805922	Mediator of RNA polymerase II transcription subunit	Med23	159090	108	9
gi|2645205	p160 myb-binding protein	Mybbp1a	152773	100	8
gi|40254124	SNF5 homolog	Snf5l1	122291	160	19
gi|124249084	EMSY protein	Emsy	135550	67	4
gi|51092285	Shugoshin-like 2	Sgol2	131850	721	34
gi|81913723	Sal-like protein 4	Sall4	114711	131	7
gi|19548750	Mediator complex subunit 24	Med24	107470	217	11
gi|1350582	MHC class II regulatory factor RFX1	Rfx1	103972	85	3
gi|25955700	PHD finger protein 14	Phf14	100497	156	13
gi|37360004	Lysine-specific histone demethylase 1A	Kdm1a	96175	114	17
gi|20177999	Mediator complex subunit 15	Med15	87462	84	5
gi|37360512	PHD finger protein 21A	Phf21a	73316	84	10
gi|19527150	Protection of telomeres protein 1	Pot1	71902	75	5
gi|8394027	Serine/threonine-protein phosphatase 2A	Ppp2ca	66079	78	17
gi|6630996	Coilin	Coil	62239	54	7
gi|13278534	Mediator complex subunit 17	Med17	64168	168	19
gi|30794412	TAF15 RNA polymerase II, TATA box binding protein (TBP)-associated factor	Taf15	58794	66	6
gi|31981077	RNA-binding protein 40	Rnpc3	58107	67	13
gi|26327831	Steroid hormone receptor ERR2	Esrrb	49023	78	17
gi|83649741	WD repeat domain 18	Wdr18	47979	114	19
gi|13278367	Rbm39 protein	Rbm39	47255	245	17
gi|1094400	CK II alpha	Csnk2a1	45185	52	11
gi|15022805	High mobility group 20A	Hmg20a	39893	72	19
gi|11528488	High mobility group 20B	Hmg20b	35962	88	14
gi|38382739	Ccnc protein	Ccnc	37241	59	8
gi|58037335	Npm2 protein	Npm2	34608	93	16

gil13385626	Mediator complex subunit 4	Med4	29820	60	14
gil12851679	centromere protein V	Cenpv	27921	329	46

Proteins that are components in known complexes are: a) PRC1; b) BCOR complex; c) E2F6.com; d) p400.com; e) MLL2. [1] Mascot protein score, [2] Fraction of the protein recovered in peptides.
