# Supplementary material for: PRC1 and PRC2 Are Not Required for Targeting of H2A.Z to Developmental Genes in Embryonic Stem Cells
Source: PLoS One. 2012 Apr 9;7(4):e34848. doi: 10.1371/journal.pone.0034848 (PMC3322156; doi:10.1371/journal.pone.0034848)
Supplement: Table S2 — Polypeptides associated with Ring1BI53A (Relates to Fig. 1). (PDF) [file pone.0034848.s007.pdf]

Supplemental Table 2. Polypeptides associated with Ring1BI53A (Relates to Fig. 1)

NCBI Acc. Nr.	Protein	Gene name	Mass (Da)	Protein Score[1]	Coverage (%)[2]
gil14028669	DNA-dependent ATPase Snf2h[a]	Snf2h	122311	54	6
gil27499606	O-linked GlcNAc transferase[a]	Ogt	118060	55	13
gil1490546	Polyhomeotic-like protein 1[a]	Phc1	106891	804	28
gil266545	Chromobox protein homolog 2[a]	Cbx2	55142	166	24
gil18043656	Mbt domain containing 1[a]	Mbtd1	53486	46	8
gil3220232	Polyhomeotic-like protein 2[a]	Phc2	47556	59	11
gil28076973	Polycomb group ring finger 6[a]	Pcgf6	40365	247	28
gil2239144	Ring finger protein 1A[a,b,c]	Ring1A	39892	559	14
**gil2239142**	**RING finger protein 1B[a,b,c]**	**Ring1B**	**38265**	**1629**	**40**
gil6678635	Polycomb group ring finger 2[a]	Pcgf2	38383	37	8
gil7304947	Chromobox homolog 8[a,b]	Cbx8	39893	85	12
gil5381327	RING1 and YY1-binding protein[a,b]	Rybp	24743	412	41
gil26329299	Polycomb group ring finger 3[a]	Pcgf3	21342	35	12
gil55958274	Polycomb complex protein Bmi1[a,b]	Bmi1	12639	44	18
gil66792788	BCL-6 interacting corepressor[b]	Bcor	190043	251	17
gil125347764	BCL6 co-repressor-like 1[b]	Bcorl1	192364	207	14
gil54607024	F-box and leucine-rich repeat protein 10[b]	Fbxl10	151401	173	11
gil148696030	MAX gene associated[c]	Mga	330371	1894	37
gil27370170	l(3)mbt-like 3[c]	L(3)mbt-like 3	100443	507	29
gil148672618	l(3)mbt-like 2[c]	L(3)mbt-like2	76153	404	26
gil17352153	E2F6a[c]	E2F6	31046	186	33
gil124486949	Transformation/transcription domain-associated protein[d]	Trrap	439911	1090	22
gil55976523	E1A-binding protein p400[d]	p400	338366	803	19
gil94421034	E1A binding protein p300[d]	p300	266044	171	9
gil737920	CREB-binding protein[d]	Cbp	268294	58	2
gil74179958	Bromodomain containing 8[d]	Brd8	95515	92	14
gil149249371	Enhancer of polycomb homolog 2[d]	Epc2	91767	97	18
gil6755382	RuvB-like protein 2[d]	Ruvbl2	51252	284	30

gi|4001805	BRG1-associated factor 53A[d]	Baf53a	47913	268	26
gi|6425087	Gamma actin-like protein[d]	Actg1	44029	249	32
gi|387090	Alpha-cardiac actin[d]	Actc	42043	259	34
gi|30425250	Actin, beta-like 2[d]	Actbl2	42319	164	16
gi|4501887	Actin, gamma 1 propeptide[d]	Actg1	42108	318	43
gi|49868	Beta-actin (aa 27-375)[d]	Actb	39446	279	38
gi|84569914	MRG-binding protein[d]	Mrgbp	22478	76	20
gi|149266757	Histone-lysine N-methyltransferase Mll2[e]	Mll2	571899	500	13
gi|145587671	Nuclear receptor coactivator 6[e]	Ncoa6	220333	52	1
gi|42734451	PAX-interacting protein 1[e]	Paxip1	120505	568	29
gi|33859492	Lysine-specific demethylase 6A[e]	Kdm6a	159240	257	23
gi|16554627	WD repeat domain 5[e]	Wdr5	37136	457	41
gi|26339944	Retinoblastoma binding protein 5[e]	Rbbp5	59745	93	15
gi|21313594	PTIP-associated 1[e]	Pa1	27820	64	22
gi|47078460	Telomere-associated protein RIF1	Rif1	267600	367	13
gi|123226656	Mediator of RNA polymerase II transcription subunit 12	Med12	244887	238	10
gi|124286862	Mediator of RNA polymerase II transcription subunit 13	Med13	241917	50	1
gi|68342456	Methylcytosine dioxygenase TET2	Tet2	215136	88	6
gi|11094397	Transcription factor 20	Tcf20	214923	607	26
gi|115270972	Mediator complex subunit 14	Med14	159966	55	2
gi|2645205	p160 myb-binding protein	Mybbp1a	152773	78	6
gi|51092285	Shugoshin-like 2	Sgol2	131850	1250	46
gi|124249084	EMSY protein	Emsy	135550	94	6
gi|1350582	MHC class II regulatory factor RFX1	Rfx1	103972	67	4
gi|25955700	PHD finger protein 14	Phf14	100497	460	24
gi|37360004	Lysine-specific histone demethylase 1A	Kdm1a	96175	256	27
gi|148698953	mCG14769		94852	90	8
gi|123212125	PHD finger protein 21A	Phf21a	71521	130	16
gi|74226948	Coilin	Coil	62700	54	3
gi|13278367	Rbm39 protein	Rbm39	47255	180	14
gi|21704096	TAR DNA-binding protein 43	Tardbp	44918	77	6
gi|12857100	High mobility group 20A	Hmg20a	39897	143	16
gi|12851679	centromere protein V	Cenpv	27921	344	46

Proteins that are components in known complexes are: a) PRC1; b) BCOR complex; c) E2F6.com; d) p400.com; e) MLL2. [1] Mascot protein score, [2] Fraction of the protein recovered in peptides.
