# Supplementary material for: PRC1 and PRC2 Are Not Required for Targeting of H2A.Z to Developmental Genes in Embryonic Stem Cells
Source: PLoS One. 2012 Apr 9;7(4):e34848. doi: 10.1371/journal.pone.0034848 (PMC3322156; doi:10.1371/journal.pone.0034848)
Supplement: Table S3 — Polypeptides associated with Ring1B in presence of 50 U Benzonase (Relates to Fig. 1). (PDF) [file pone.0034848.s008.pdf]

Supplemental Table 3. Polypeptides associated with Ring1B in presence of 50U Benzonase (Relates to Fig. 1)

NCBI Acc. Nr.	Protein	Gene name	Mass (Da)	Protein Score[1]	Coverage (%)[2]
gil110835733	Polyhomeotic-like 1[a]	Phc1	101191	189	20 %
gil28076973	Polycomb group ring finger 6[a]	Pcgf6	40365	128	20 %
**gil26354897**	**RING finger protein 1B[a,b,c]**	**Ring1B**	**38107**	**709**	**41 %**
gil5381327	RING1 and YY1-binding protein[a,b]	Rybp	24743	199	36 %
gil27085286	BCL-6 interacting corepressor isoform[b]	Bcor	194094	61	1 %
gil6692607	MAX gene associated[c]	Mga	330371	288	11 %
gil27734414	l(3)mbt-like 2[c]	L(3)mbt-like2	80045	51	5 %
gil124486949	Transformation/transcription domain-associated protein[d]	Trrap	439911	757	18 %
gil27348237	E1A-binding protein p400[d]	Ep400	334589	309	8 %
gil27532963	Enhancer of polycomb homolog 1[d]	Epc1	90925	44	4 %
gil4001805	BRG1-associated factor 53A[d]	Baf53a	47913	138	20 %
gil809561	Gamma actin-like protein[d]	Actg1	44029	104	20 %
gil30425250	Actin, beta-like 2[d]	Actbl2	42319	76	10 %
gil149266757	Histone-lysine N-methyltransferase Mll2[e]	Mll2	571899	72	3 %
gil33859492	Lysine-specific demethylase 6A[e]	Kdm6a	159240	65	5 %
gil42734451	PAX-interacting protein 1[e]	Paxip1	120505	118	10 %
gil26339944	Retinoblastoma binding protein 5[e]	Rbbp5	59745	95	7 %
gil16554627	WD repeat domain 5[e]	Wdr5	37136	132	27 %
gil21313594	PTIP-associated 1[e]	Pa1	27820	50	11 %
gil3426322	Mediator of RNA polymerase II transcription subunit 12	Med12	234343	86	5 %
gil115270972	Mediator complex subunit 14	Med14	159966	60	2 %
gil51092285	Shugoshin-like 2	Sgol2	131850	105	16 %
gil18921439	Coiled-coil and C2 domain containing 1a	Cc2d1a	66661	55	1 %
gil11528488	High mobility group 20B	Hmg20b	35962	53	8 %

Proteins that are components in known complexes are: a) PRC1; b) BCOR complex; c) E2F6.com; d) p400.com; e) MLL2. [1] Mascot protein score, [2] Fraction of the protein recovered in peptides.
