# Supplementary material for: PRC1 and PRC2 Are Not Required for Targeting of H2A.Z to Developmental Genes in Embryonic Stem Cells
Source: PLoS One. 2012 Apr 9;7(4):e34848. doi: 10.1371/journal.pone.0034848 (PMC3322156; doi:10.1371/journal.pone.0034848)
Supplement: Table S4 — Polypeptides associated with p400 (Relates to Fig. 1). (PDF) [file pone.0034848.s009.pdf]

Supplemental Table 4. Polypeptides associated with p400 (Relates to Fig. 1)

NCBI Acc. Nr.	Protein	Gene name	Mass (Da)	Protein Score[1]	Coverage (%)[2]
gil148687065	Transformation/transcription domain-associated protein	TRRAP	420980	165	6
**gil27348237**	**E1A-binding protein p400**	**p400**	**334589**	**146**	**5**
gil149268663	Similar to Kinesin-like protein Kifc1	Kifc1-like	89770	458	30
gil6679655	Enhancer of polycomb homolog 1	Epc1	85228	87	5
gil39795654	Malignant Brain Tumor domain-containing 1	Mbtd1	70923	66	8
gil12805675	Dnmt1 associated protein-1	Dmap1	51543	56	23
gil4001805	Actin-like 6A	Baf53a	47913	202	15
gil809561	Gamma-actin	Actg	41335	104	25

[1] Mascot protein score, [2] Fraction of the protein recovered in peptides.
