# Supplementary material for: PRC1 and PRC2 Are Not Required for Targeting of H2A.Z to Developmental Genes in Embryonic Stem Cells
Source: PLoS One. 2012 Apr 9;7(4):e34848. doi: 10.1371/journal.pone.0034848 (PMC3322156; doi:10.1371/journal.pone.0034848)
Supplement: Table S5 — Primers for ChIP analysis (Relates to Figs. 2 and 5). (PDF) [file pone.0034848.s010.pdf]

Supplemental Table 5. Primers for ChIP analysis (Relates to Figs. 2 and 5)

Promoter	Oligo name	Sequence	Ref.
Hoxb1	Hoxb1promfw	TTAGCCCATTGGCCTGGGAGAGAT	[1]
	Hoxb1promrev	TGAAGCTTGAGCTTGAGCCCATGGCCCG	[1]
Hoxb13	Hoxb13promfw	ATGAGCCTCTCTCCCCCAGG	[1]
	Hoxb13promrev	AATCGCTCCCAGCTCGAACGG	[1]
Hoxd10	Hoxd10prof	TAGTAGATGTCGCTGTTGTCCG	[1]
	Hoxd10pror	ACATGACAACCAAGCCAATGAGA	[1]
Oct-4	Pou5f1profor	GCTGGCGGAAAGACACTAAG	
	Pou5f1prorev	CAGAGCATGGTGTAGGAGCA	
Nanog	NanogBprofor	CTATCGCCTTGAGCCGTTGG	[2]
	NanogBprorev	AACTCAGTGTCTAGAAGGAAAGATCA	[2]
Cdx2	Cdx2promoterfor	GGACTCCGCGAGCCAA	[3]
	Cdx2promoterrev	CTCAGCCCACGGTGCTC	[3]
Gata4	Gata4promoterF	AAGAGCGCTTGCGTCTCTA	[3]
	Gata4promoterR	TTGCTAGCCTCAGATCTACGG	[3]

1. Eskeland R, Leeb M, Grimes GR, Kress C, Boyle S, et al. (2010) Ring1B compacts chromatin structure and represses gene expression independent of histone ubiquitination. Mol Cell 38: 452-464.
2. O'Neill LP, VerMilyea MD, Turner BM (2006) Epigenetic characterization of the early embryo with a chromatin immunoprecipitation protocol applicable to small cell populations. Nat Genet 38: 835-841.
3. Stock JK, Giadrossi S, Casanova M, Brookes E, Vidal M, et al. (2007) Ring1-mediated ubiquitination of H2A restrains poised RNA polymerase II at bivalent genes in mouse ES cells. Nat Cell Biol 9: 1428-1435.
