# Supplementary material for: PRC1 and PRC2 Are Not Required for Targeting of H2A.Z to Developmental Genes in Embryonic Stem Cells
Source: PLoS One. 2012 Apr 9;7(4):e34848. doi: 10.1371/journal.pone.0034848 (PMC3322156; doi:10.1371/journal.pone.0034848)
Supplement: Table S6 — Antibodies used for this study. (DOC) [file pone.0034848.s011.doc]

Supplemental Table 6. Antibodies used for this study

Antibody against	Clone	Working dilution for Westerns	Origin
H2A.Z	ab4174	(1:1000)	Abcam
H2A	07-146	(1:1000)	Millipore
H3K27me3	07-449	(1:2000)	Millipore
H3K4me3	07-473	(1:2000)	Millipore
H3	ab-1791	(1:50000)	Abcam
EZH2	07-689	(1:2000)	Millipore
Ring1B	D139-3	(1:1000)	MBL
DMAP1	ab2848	(1:500)	Abcam
p400	A300-541A-1	(1:2000)	Bethyl Laboratories, Inc.
Mll2	CS-099-100	(1:500)	Diagenode
Menin	A300-114A	(1:1000)	Bethyl Laboratories, Inc.
Rybp	AB3637	(1:1000)	Millipore
Suz12	CS-029-100	(1:1000)	Diagenode
HP1α	2G9	(1:1000)	Euromedex
Tubulin	T9026	(1:1000)	Sigma
